# Emergence of *mcr-8.1*-bearing MDR-hypervirulent *Klebsiella pneumoniae* ST307

**DOI:** 10.1128/spectrum.01910-24

**Published:** 2024-12-13

**Authors:** Jie Sheng, Rory Cave, Mary M. Ter-Stepanyan, Siyu Lu, Yingxiong Wang, Taihang Liu, Hermine V. Mkrtchyan

**Affiliations:** 1 School of Basic Medical Sciences, Chongqing Medical University, Chongqing, China; 2 The Joint International Research Laboratory of Reproduction and Development, Ministry of Education, Chongqing, China; 3 School of Biomedical Sciences, University of West London, London, United Kingdom; 4 Department of Epidemiology, Faculty of Public Health, Yerevan State Medical University after M. Heratsi, Yerevan, Armenia; 5 Research Center of Maternal and Child Health Protection, Yerevan, Armenia; JMI Laboratories, North Liberty, Iowa, USA

**Keywords:** *Klebsiella pneumoniae*, whole genome sequencing, multidrug resistance, *mcr-8.1*, hypervirulent

## Abstract

We report for the first time whole-genome sequencing of four multidrug-resistant sequence type (ST) 307 *Klebsiella pneumoniae* recovered from patients in two hospitals in Armenia. Comparative genomic analysis revealed that the isolates were closely related, with a maximum of 39 single nucleotide polymorphism (SNP) differences in the core genome. All Armenian isolates carried the integrative and conjugative element ICE*Kp*4, which bears the yersiniabactin locus, and shared a common evolutionary origin, diverging around 2005 (95% CI: 1999 to 2011). Antibiotic susceptibility testing showed resistance to several antibiotics, including ampicillin, amoxicillin-clavulanic acid, cefepime, ceftazidime, norfloxacin, levofloxacin, and chloramphenicol. Specifically, isolates designated as ARM03 and ARM06 were resistant to piperacillin-tazobactam, ARM04 and ARM05 had intermediate resistance to both piperacillin-tazobactam and imipenem, and ARM03 showed intermediate resistance to amikacin. We further identified antimicrobial resistance (AMR) genes in four Armenian isolates, including *bla*_OXA-1_, *bla*_TEM-1D_, *bla*_SHV-28_, *dfrA14*, *tet(A*), *sul2*, *qnrB1*, *aac(6´)-Ib-cr*, *strA*, *strB* and the extended-spectrum β-lactamase gene *bla*_CTX-M-15_. Additionally, ARM03 and ARM06 also obtained *dfrA5*, *sul1*, *sul3*, *cmlA1*, *mphA*, *aph3-Ia* and the unique colistin resistance gene *mcr-8.1*, which was absent in all other publicly available ST307 isolates. These two isolates also acquired aerobactin siderophore-encoding gene clusters (*iucABCD-iutA*) and the hypermucoidy locus *rmpADC* (ARM06 had *rmpA* fragment). ARM04 and ARM05, as well as ARM03 and ARM06, had nearly identical AMR and virulence genes, along with similar plasmid replicon profiles, respectively. Our findings suggest that a transmission event occurred between the two hospitals in Armenia, likely facilitated by patients or community members, during which *K. pneumoniae* ST307 isolates acquired plasmids carrying AMR and virulence genes.

**IMPORTANCE:**

Multidrug-resistant (MDR) *Klebsiella pneumoniae* sequence type (ST) 307 has emerged as a high-risk clone associated with hospital- and community-acquired infections, posing a major threat to global public health. We report in-depth comparative genomics analyses of *K. pneumoniae* ST307 isolates recovered from patients in Armenia. The unique colistin resistance gene *mcr-8.1* identified in ARM03 and ARM06 was absent in all other ST307 isolates obtained from the publicly available data sets. ARM03 and ARM06 also acquired aerobactin siderophore-encoding gene clusters (*iucABCD-iutA*) and the hypermucoidy locus *rmpADC* (ARM06 possessed incomplete *rmpA* fragment). Our findings suggest that a transmission event has occurred between two hospitals in Armenia either through patients or community members. In addition, the Armenian isolates obtained plasmids carrying virulence and AMR genes during the transmission event. Our study emphasises the importance of genomic surveillance of this emerging MDR-hypervirulent pathogen to provide early interventions.

## INTRODUCTION

The *Klebsiella pneumoniae* sequence type (ST) 307 was first recorded in the Netherlands in 2008 (*Kp* MLST database) (1). Since then, *K. pneumoniae* ST307 has been isolated from various sources, including humans (2, 3), pets (4, 5), wild animals (6), and the environment (7). This sequence type is increasingly recognized as a significant high-risk clone worldwide, with notable outbreaks reported in Europe and the USA (8, 9). Known for its extensive drug resistance, *K. pneumoniae* ST307 also exhibits enhanced virulence factors such as hypermucoviscosity and enhanced iron acquisition, contributing to its persistence and spread in healthcare settings (8). Besides sharing a feature of multidrug resistance, most ST307 isolates exhibited the same K and O loci (KL102 and O2v2) (8) and had a high prevalence of *bla*_CTX-M_ genes, with *bla*_CTX-M-15_ being the most common extended-spectrum β-lactamase (ESBL)-encoding gene (10). A recent global study found that the *bla*_CTX-M-15_ was detected in 93.7% (89/95) of *K. pneumoniae* ST307 genomes analyzed (1). ST307 is particularly concerning due to its association with severe infections and its high resistance to multiple antibiotics, which significantly limits treatment options. It frequently causes bloodstream infections, pneumonia, urinary tract infections, and other serious healthcare-associated infections (11–13). The increased virulence and resistance of ST307 necessitate enhanced surveillance and stringent infection control practices to manage its spread and impact on public health.

Mobile genetic elements (MGEs) play important roles in the acquisition and dissemination of antimicrobial resistance (AMR) genes and virulence determinants (14). The MGEs include insertion sequences, transposons, integrons, plasmids, integrative and conjugative elements, prophages, and genomic islands (15). Through horizontal gene transfer, MGEs substantially facilitate the formation of multidrug-resistant (MDR) isolates and the spread of AMR genes and virulence determinants among different bacteria, resulting in genomic diversification (16, 17). The integrative and conjugative element ICE*Kp*, located on the accessory genome (18), is the most common virulence-associated mobile genetic element in *K. pneumoniae* and can mobilize the spread of yersiniabactin (*ybt*) locus (19). Comparative analysis of 2,498 *K*. *pneumoniae* genomes showed that the *ybt* locus was detected in 40% of *K. pneumoniae* genomes and was further grouped into 17 distinct *ybt* phylogenetic lineages, each associated with 1 of 14 different ICE*Kp* structural variants (ICE*Kp*1–ICE*Kp*14) (20). MDR *K. pneumoniae* and hypervirulent *K. pneumoniae* (hv*KP*) are generally associated with two distinct subsets of clonal lineages (21). Some typical virulence determinants (e.g., *rmpA*, *iuc*, and *iro*) were widely used for the identification of hv*KP* from classical *K. pneumoniae* (22, 23). MDR-hypervirulent *K. pneumoniae* are simultaneously hypervirulent and resistant to multiple antibiotics. This was commonly considered as the result of classical MDR *K. pneumoniae* lineages acquiring virulence determinants, hv*KP* acquiring MDR phenotypes, or *K. pneumoniae* acquiring plasmids containing both AMR genes and hypervirulence determinants (24). The prevalence of MDR-hypervirulent *K. pneumoniae* has increased over the past decade (25, 26). Recent reports include five cases of fatal outcomes for hospital patients due to infections caused by carbapenem-resistant hv*KP* isolates (27); an outbreak of ST11 carbapenem-resistant MDR hv*KP* isolates in a Chinese hospital also resulted in fatalities (28). More recently, six colistin-resistant MDR *K. pneumoniae* isolates (belonging to ST11, ST5253, and ST86) harboring hypervirulent biomarker genes (*peg344*, *iroB*, *iucA*, *rmpA*, and *rmpA2*) have been identified (29). The rise in severe infections and the increasing limitations of effective treatments make MDR-hypervirulent *K. pneumoniae* a true superbug that poses a serious threat to public health (30). Armenia is a low-and middle-income country where genomic surveillance of Gram-negative bacteria is scarce. To the best of our knowledge, this is the first report of whole-genome sequencing and analysis of *K. pneumoniae* ST307 recovered from patients in Armenia. Here, we report in-depth comparative genomic analyses of *K. pneumoniae* ST307 isolates, which provide important insights into the genetic variation and evolution of *K. pneumoniae* ST307 circulating in clinical settings in Armenia. Our findings inform the need for interventions for better diagnostic and infection prevention and control capacity to reduce the spread of this hypervirulent clone and inform effective treatment strategies.

## RESULTS

### Isolates and antibiotic susceptibility testing

Four out of the eight *K. pneumoniae* previously reported isolates (31) included in this study belonged to ST307. ARM03, ARM05, and ARM06 were recovered from different patients (throat and urine samples) in the same hospital (H2), whereas ARM04 was recovered from a sputum sample in hospital H1 (Table S1). All isolates were resistant to 8 (*n* = 1) and 9 (*n* = 3) of the 11 antibiotics tested. All four isolates were resistant to the aminopenicillin antibiotic ampicillin, β-lactam antibiotic amoxicillin-clavulanic acid, cephalosporin antibiotics cefepime and ceftazidime, fluoroquinolone antibiotics norfloxacin and levofloxacin, as well as chloramphenicol, but were sensitive to carbapenem antibiotic meropenem (Table S1). In addition, ARM03 and ARM06 were resistant to the β-lactam antibiotic piperacillin-tazobactam, whereas ARM04 and ARM05 had intermediate resistance to piperacillin-tazobactam and carbapenem antibiotic imipenem. Furthermore, ARM03 was found to have an intermediate resistance to the aminoglycoside antibiotic amikacin. Although ARM04 and ARM05 were recovered from two different patients in two different hospitals (H1 and H2), they exhibited similar antibiotic resistance profiles to all tested antibiotics. In addition, ARM03 and ARM06 recovered from two different patients in the same hospital (H2) had nearly identical resistance profiles, with the only difference being that ARM03 showed intermediate resistant to amikacin (Table S1).

### Phylogenetic analysis of global *K. pneumoniae* ST307 population

**Fig 1:**
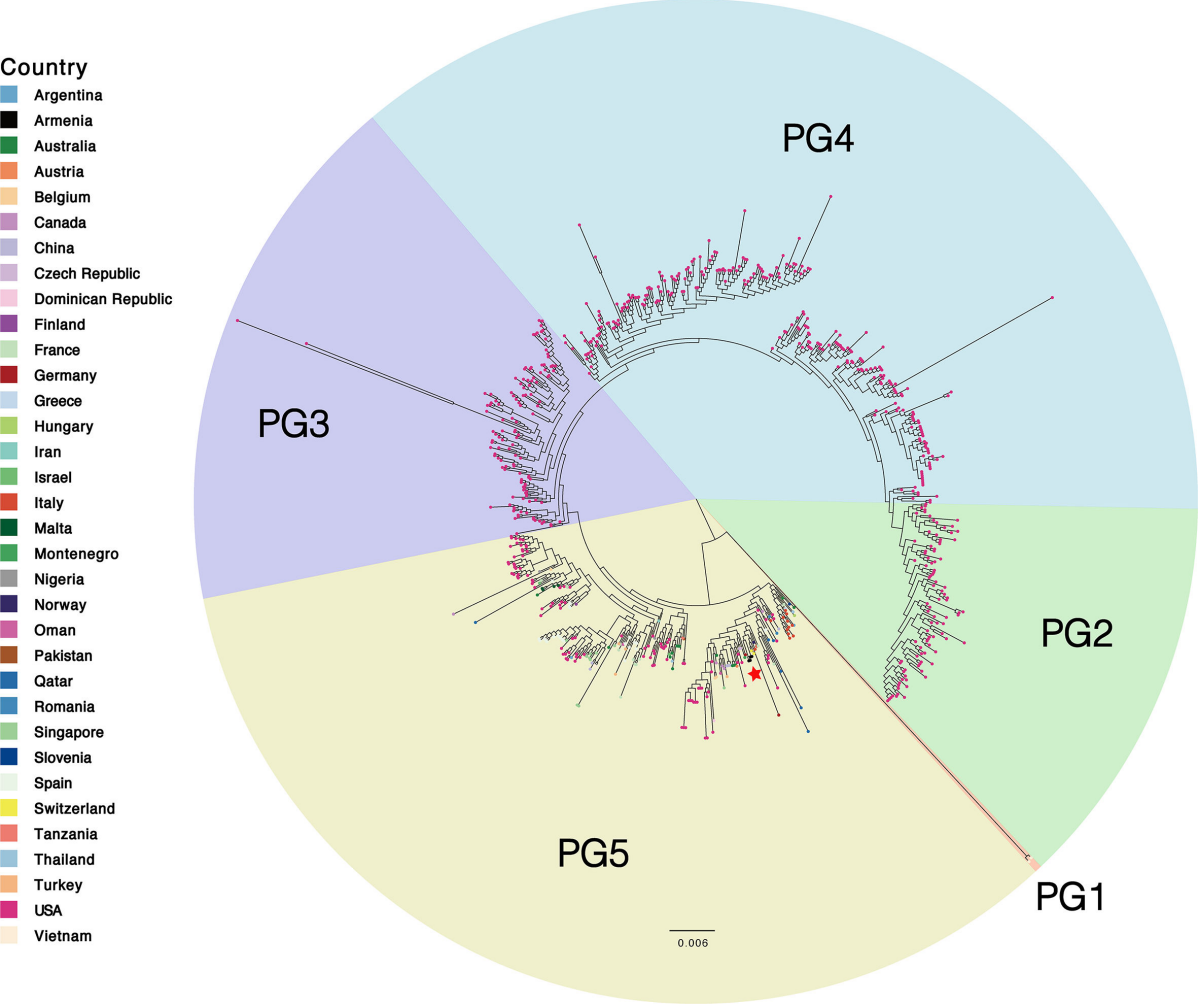
Core SNP phylogenetic analysis of global *K. pneumoniae* ST307 isolates. The evolutionary tree is divided into five phylogenetic groups according to the FastBaps software and is labeled with different colors. The color of the circle at the end of each branch represents the country where the strain was collected. The Armenian isolates are marked with red stars.

To investigate the genetic relatedness and differences of the Armenian isolates with those ST307 isolates already deposited in public data sets, we downloaded 745 *K*. *pneumoniae* ST307 genomes from the Pathogenwatch database (Table S2) and conducted a core single nucleotide polymorphism (SNP) phylogenetic analysis. All genomes were previously recovered from 34 countries and four different animal sources, most of which (684/749) belonged to capsule type K102 and O antigen type O2 (the remaining were unknown) (Table S2). To infer the genetic structure based on shared patterns of sequence variation, the evolutionary tree of the *K. pneumoniae* ST307 was partitioned into five phylogenetic groups (PGs) using FastBaps v.1.0.4, which was based on the hierarchical Bayesian clustering algorithm (Fig. 1). PG1 included two isolates recovered from Vietnam in 2015 and 2016, respectively. PG2, PG3, and PG4 contained 95, 126, and 274 isolates recovered from human sources in America, respectively. PG5 included a total of 252 isolates, including our isolates recovered from Armenia (Table S3).

**Fig 2:**
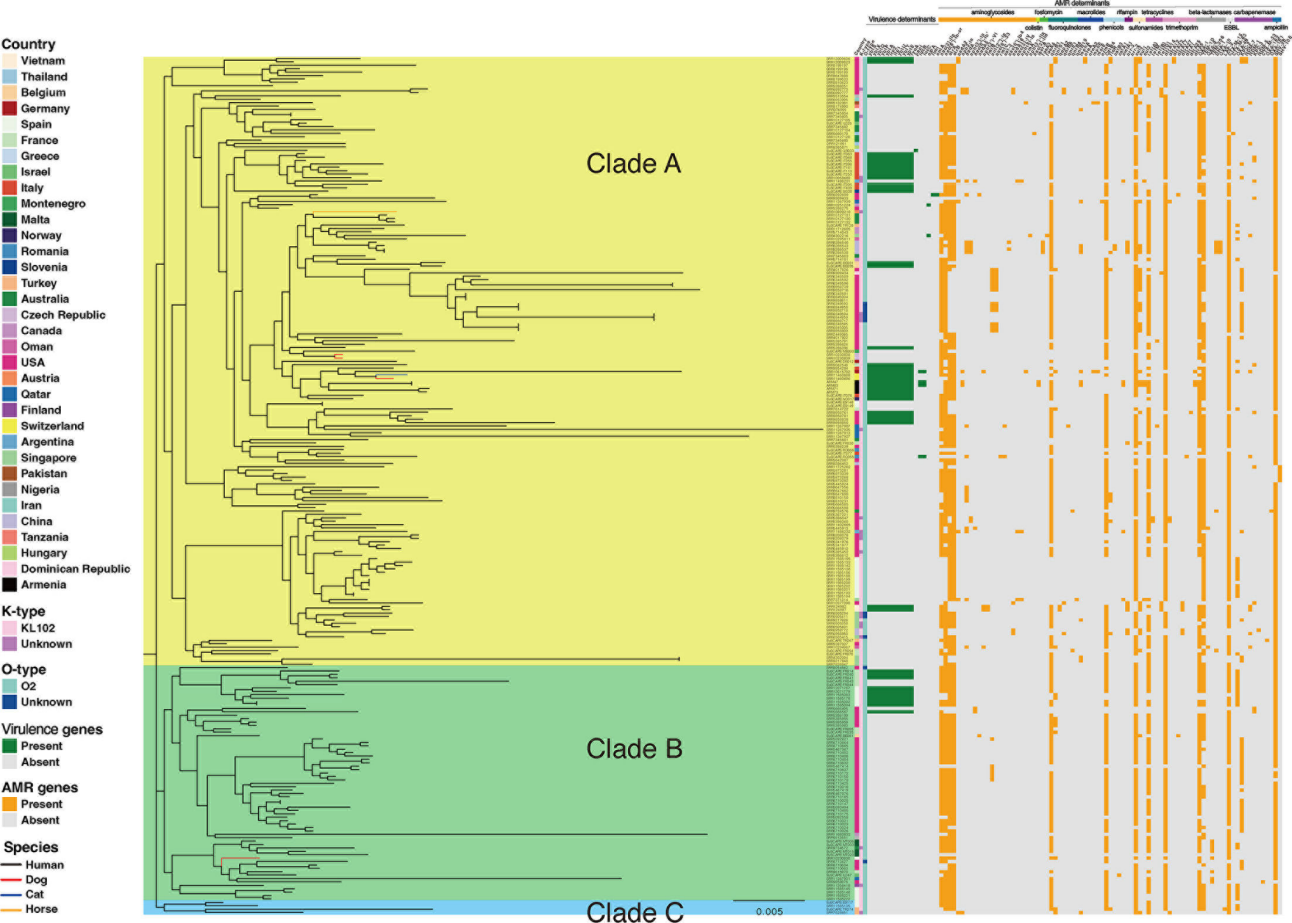
*K. pneumoniae* ST307 PG5 core maximum likelihood phylogenetic tree, along with a heatmap of virulence and AMR genes. The phylogenetic tree has three distinct clades, indicated by color. Line color represents host species. The heatmap columns are as follows: (i) country; (ii) capsule (K) serotype; (iii) lipopolysaccharide (LPS) O-antigen serotype; (iv) virulence determinants (dark green: present, gray: absent), and (v) AMR genes (orange: present, gray: absent).

PG5 contained isolates recovered from 34 countries and four different sources (human, dog, cat, and horse). Based on their phylogenetic relationship, *K. pneumoniae* ST307 PG5 isolates were divided into three main clades (Fig. 2). However, the clade division does not appear to be related to a particular country or source. The *K. pneumoniae* ST307 isolates recovered in our study clustered together in clade A and were phylogenetically closely related to each other, with a maximum SNP distance of 39. There was no SNP difference between ARM03 and ARM06, while there were only three SNP differences between ARM04 and ARM05 (Table S4). The Armenian isolates were found to be closely related to a phylogenetic clade which consists of four isolates including one recovered from a human in Italy (SRR9854284, 2019), one from a human in Germany (SRR10615702, 2019), and two from Switzerland, one from a dog (SRR11460696, 2020) and one from a cat (SRR11460688, 2020).

### Origins of *K. pneumoniae* ST307 PG5

**Fig 3:**
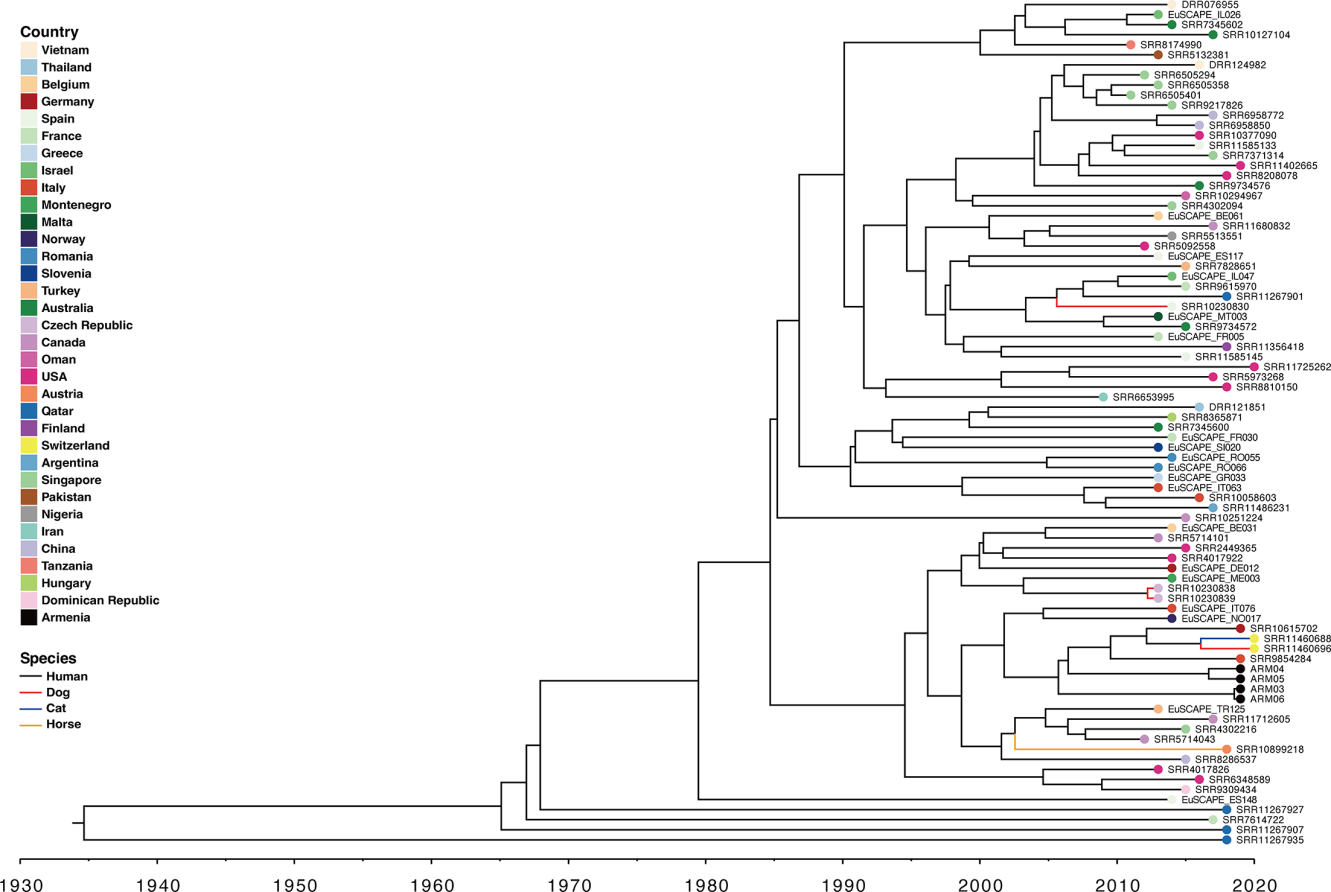
Bayesian evolution analysis of *K. pneumoniae* ST307 PG5 isolates. The color of the circle at the end of each branch represents the country where the isolate was collected. Line color represents the host species.

We further conducted an in-depth analysis of isolates in PG5, including those recovered from Armenia (included in this study). Repetitive sequences from the same host species, country, and collection date were clustered at 99% identity using CD-HIT to remove redundancy. A total of 84 non-redundant strains were used to reconstruct the maximum clade credibility (MCC) phylogenetic tree to infer the evolutionary origins of the Armenian isolates with those phylogenetically closely related ST307 isolates belonging to PG5 (Fig. 3). The ST307 PG5 MCC tree had an estimate substitution rate of 8.414 × 10^−4^ substitutions per site per year (95% CI: 6.104 × 10^−4^ to 1.0779 × 10^−3^) and an inferred tree root date of 1934 (95% CI: 1855 to 1990). The most recent inferred date of divergence of the Armenian isolates to their closest phylogenetically related isolates (SRR9854284, SRR10615702, SRR11460696, and SRR11460688) was 2005 (95% CI: 1999 to 2011), indicating that they shared and had descended from a common ancestor. The most recent divergence date that was shared by both the ARM04 and ARM05 was 2016 (95% CI: 2014 to 2018), while the most recent divergence date between ARM03 and ARM06 was 2018 (95% CI: 2017 to 2019). This indicates a closer phylogenetic relatedness between the isolates of ARM03 and ARM06, as well as between ARM04 and ARM05.

### Genotypic characterization of antibiotic resistance

The AMR gene profiles of the Armenian *K. pneumoniae* ST307 were compared to the remaining 248 *K*. *pneumoniae* ST307 isolates belonging to PG5. We identified a total of 81 different AMR genes within the resistome of the PG5 isolates, with the average number of AMR genes per isolate being 12 (ranging between 2 and 21). (Fig. 2; Table S5). A number of AMR genes, including β-lactam resistance genes *bla*_OXA-1_ (207/252) and *bla*_TEM-1D_ (184/252), aminoglycoside resistance genes *aac(3)-IIa* (185/252), *aac(6´)-Ib-cr* (205/252), *strA* (201/252), and *strB* (202/252), fluoroquinolone resistance gene *qnrB1* (197/252), phenicol resistance gene *CatB4* (198/252), sulfonamide resistance gene *sul2* (223/252), tetracycline resistance gene *tet(A)* (179/252), trimethoprim resistance gene *dfrA14* (234/252), ESBL-encoding gene *bla*_CTX-M-15_ (232/252), and ampicillin resistance gene *bla*_SHV-28_ (241/252), were detected in the majority of the PG5 isolates, suggesting that multidrug resistance was preserved in *K. pneumoniae* ST307 isolates belonging to PG5 (Fig. S1; Table S5). In addition, two CTX-M-type ESBL genes (*bla*_CTX-M-15_ and *bla*_CTX-M-63_) were identified in PG5 isolates, with *bla*_CTX-M-15_ (232/252) being the most common ESBL-encoding gene, also found in all Armenian isolates. Moreover, the ESBL-encoding gene *bla*_CTX-M-63_ was only detected in SRR5660179 recovered from a patient in Thailand in 2016.

The genomic characterization of the Armenian isolates revealed that all four isolates carried multiple genes conferring resistance to a number of antibiotics (Table 1). Interestingly, ARM04 and ARM05 had identical AMR gene profiles (11 genes), including β-lactam resistance genes *bla*_OXA-1_ and *bla*_TEM-1D_, ESBL-encoding gene *bla*_CTX-M-15_, ampicillin resistance gene *bla*_SHV-28_, trimethoprim resistance gene *dfrA14*, tetracycline resistance gene *tet(A*), sulfonamide resistance gene *sul2*, fluoroquinolone resistance gene *qnrB1*, and aminoglycoside resistance genes *aac(6´)-Ibcr*, *strA*, and *strB*. In addition to the above 11 AMR genes, ARM03 and ARM06 possessed the trimethoprim resistance gene *dfrA5,* sulfonamide resistance genes *sul1* and *sul3*, chloramphenicol resistance gene *cmlA1*, macrolide resistance gene *mphA*, aminoglycoside resistance gene *aph3-Ia*, and colistin resistance gene *mcr-8.1*. Eighteen AMR genes were simultaneously detected both in ARM03 and ARM06, which was greater than the average number of antibiotic resistance genes (*n* = 12), compared to the *K. pneumoniae* ST307 isolates in PG5. The colistin resistance gene *mcr-8.1* was unique to ARM03 and ARM06 isolates and was absent in all other ST307 isolates obtained from the Pathogenwatch database.

**TABLE 1:** Genomic characteristics of four Armenian isolates^*a*^

	ARM03	ARM04	ARM05	ARM06
β-Lactams	OXA-1;TEM-1D	OXA-1;TEM-1D	OXA-1;TEM-1D	OXA-1;TEM-1D
ESBLs	CTX-M-15	CTX-M-15	CTX-M-15	CTX-M-15
Ampicillin	SHV-28	SHV-28	SHV-28	SHV-28
Trimethoprim	*dfrA14*; *dfrA5*	*dfrA14*; *dfrA5*	*dfrA14*; *dfrA5*	*dfrA14*; *dfrA5*
Tetracycline	*tet(A)*	*tet(A)*	*tet(A)*	*tet(A)*
Sulfonamides	*sul1*; *sul2*; *sul3*	*sul2*	*sul2*	*sul1*; *sul2*; *sul3*
Phenicols	*cmlA1*	−	−	*cmlA1*
Macrolides	*mphA*	−	−	*mphA*
Fluoroquinolones	*qnrB1*	*qnrB1*	*qnrB1*	*qnrB1*
Colistin	*mcr-8.1*	−	−	*mcr-8.1*
Aminoglycosides	*aac(6´)-Ib-cr*; *aph3-Ia*; *strA*; *strB*	*aac(6´)-Ib-cr*; *strA*; *strB*	*aac(6´)-Ib-cr*; *strA*; *strB*	*aac(6´)-Ib-cr*; *aph3-Ia*; *strA*; *strB*
Hypermucoidy (*rmpA* and/or *rmpA2*)	*rmpA*; *rmpC*; *rmpD*	−	−	*rmpA* (fragment); *rmpC*; *rmpD*
Yersiniabactin (*ybt*)	*irp1*; *irp2*; *ybtAEPQSTUX*; *fyuA*	*irp1*; *irp2*; *ybtAEPQSTUX*; *fyuA*	*irp1*; *irp2*; *ybtAEPQSTUX*; *fyuA*	*irp1*; *irp2*; *ybtAEPQSTUX*; *fyuA*
Aerobactin (*iuc*)	*iucABCD-iutA*	−	−	*iucABCD-iutA*
MLST	ST307	ST307	ST307	ST307
Capsule (K) serotype	K102	K102	K102	K102
lipopolysaccharide (LPS) O-antigen serotype	O2	O2	O2	O2
Plasmid replicon	IncFIB(K); IncFIB(Mar); IncFII(K); IncHI1B	IncFIB(K)	IncFIB(K)	IncFIB(K); IncFIB(Mar); IncFII(K); IncHI1B
ICEs	ICE*Kp*4	ICE*Kp*4	ICE*Kp*4	ICE*Kp*4

^*a*^ ICEs, integrative and conjugative elements; −, negative. Hypermucoidy (*rmpA* and/or *rmpA2*) represents the regulator of mucoid phenotype A.

### Accessory genome and unique genes

To further investigate the unique genomic characteristics of the Armenian isolates, we performed pangenome analysis of all ST307 isolates found in PG5. The pangenome comprised a total of 14,612 genes, including 4,318 core genes and 10,294 accessory genes. The Pearson correlation heatmap showed that four Armenian isolates shared many accessory genes, with Pearson correlation coefficient range of 0.7052–0.9893, while the mean Pearson correlation coefficient was 0.641 (SD = 0.1299, range: 0.2455 to 1) for all isolates in PG5 (Fig. S2). ARM03 had a significantly high correlation in its accessory genome with ARM06 (*r* = 0.9893, Z score = 6.57, *P* < 2.2 × 10^−16^), whereas ARM04 represented a strong correlation with ARM05 (*r* = 0.9885, Z score = 4.54, *P* < 2.2 × 10^−16^).

Using Scoary, which calculates the associations between all genes in the accessory genome and specific traits (32), we identified two genes, *oadB* and *yeeO*, that were exclusively present in all four Armenian ST307 isolates (Table S6). As an extremely hydrophobic integral membrane protein, *oadB* catalyzes the decarboxylation of oxaloacetate coupled to Na+ translocation (33, 34). *yeeO* is a multidrug efflux transporter of flavin adenine dinucleotide (FAD) and flavine mononucleotide (FMN) (35). Moreover, 13 genes identified as unique to ARM03 and ARM06 had known functions, including modulating protein (*ymoA*), isonitrile hydratase (*inhA*), phosphoethanolamine lipid A transferase (*mcr-8.1*), transcriptional activator protein (*copR*), adaptive-response sensory-kinase (*sasA*), diacylglycerol kinase (*dgkA*), DNA gyrase inhibitor (*sbmC*), D-aminopeptidase (*dap*), cardiolipin synthase B (*clsB*), heme-binding protein A (*hbpA*), IS1182 family transposase ISCfr1, IS3 family transposase ISKpn11, and glucose 1-dehydrogenase (Table S6). We identified that 8 out of these 13 genes (*ymoA*, *inhA*, *mcr-8.1*, *copR*, *sasA*, *dgkA*, *sbmC*, and *dap*) were located within a 16,409 bp genomic fragment. Using BLAST search analysis, we found that this genomic fragment was part of the *K. pneumoniae* plasmid pKP57-mcr8 (NCBI accession number CP088130.1, aligned length: 16,409 bp, coordinates: 5,332 bp–21,740 bp) with 100% query coverage and 100% similarity. Therefore, we can hypothesize that ARM03 and ARM06 may come to possess the colistin resistance gene *mcr-8.1* through a plasmid carrying this gene.

### Comparative analysis of virulence genes

PG5 encompassed a total of 252 isolates, with no virulence genes detected in 207 of them. However, the remaining 45 PG5 isolates contained a total of 19 virulence genes (Fig. 2; Table S5). This includes EuSCAPE_RO055, isolated from a human in Romania in 2014, which harbored five aerobactin siderophore-encoding operons (*iucABCD-iutA*). Forty-one isolates, including ARM04 and ARM05, possessed 11 yersiniabactin genes (*irp1*, *irp2*, *ybtAEPQSTUX*, and *fyuA*). Furthermore, three isolates (ARM03, ARM06, and SRR10615702) were identified with 11 yersiniabactin genes, five aerobactin siderophore-encoding operons, and three hypermucoidy locus *rmpADC* (ARM06 has *rmpA* fragment). Additionally, the virulence genes detected in ARM03, ARM06, and SRR10615702 belonged to *ybt* locus sequence type 10 (*ybt10*), aerobactin lineage *iuc1*, and *rmp* sequence type 1 (*rmp1*), with ARM06 carrying an incomplete *rmp1*. Previous studies demonstrated that both the *iuc1* and *rmp1* were present in IncFIB(K) virulence plasmid (36, 37). We further conducted a BLAST search on the 18,078bp and 18,147bp genomic regions where five aerobactin siderophore-encoding operons were located in ARM03 and ARM06, respectively. The result showed that they all showed 100% sequence coverage and 99.99% sequence identity with the plasmid pK2044 (NCBI accession number: AP006726.1) found in *K. pneumoniae* NTUH-K2044. Besides, *rmpA* was also found to present on the plasmid pK2044. Thus, we hypothesize that ARM03 and ARM06 may have acquired a plasmid carrying the *iuc* and *rmp*.

### Integrative and conjugative element ICE*Kp*

**Fig 4:**
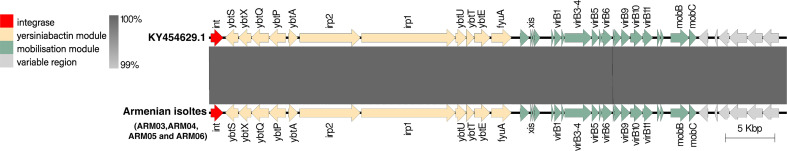
Comparison of the structures of the integrative and conjugative element ICE*Kp*4. The sequence of the 58,199 bp ICE*Kp*4 region from all four Armenian isolates was aligned against the ICE*Kp*4 sequence from *K. pneumoniae* strain 16703568 (GenBank accession number: KY454629.1, coordinates: 1–58,199 bp). Arrows represent open reading frames.

The yersiniabactin gene cluster is often mobilized by the integrative and conjugative element ICE*Kp*, which can be further divided into different structural variants according to *ybt* locus typing (20). A total of six ICE*Kp* types (ICE*Kp*2, ICE*Kp*3, ICE*Kp*4, ICE*Kp*5, ICE*Kp*11, and ICE*Kp*12) were identified in all isolates belonging to PG5 (Fig. S3). ICE*Kp*4 was detected in all four Armenian isolates, as well as in their closest phylogenetically related clades (SRR9854284, SRR10615702, SRR11460696, and SRR11460688). The total length of this ICE*Kp*4 sequence identified in all Armenian isolates was 58,199 bp, which had 100% query coverage and 99.97% similarity with the ICE*Kp*4 sequence (NCBI accession number: KY454629.1, total length 58,199 bp) detected in *K. pneumoniae* 16703568 (Fig. 4).

### Plasmid typing

**Fig 5:**
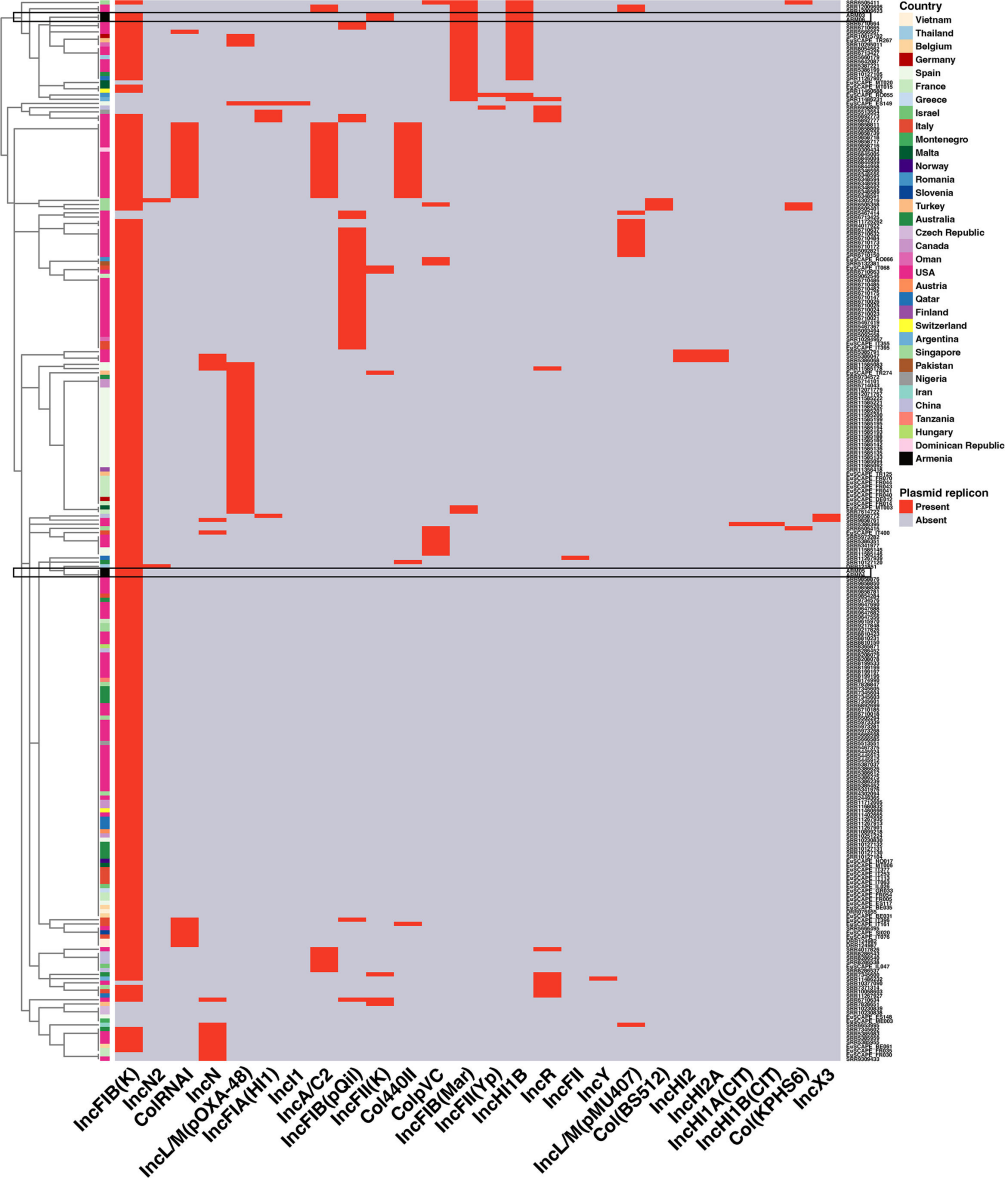
The plasmid replicon profiles of *K. pneumoniae* ST307 PG5 isolates (red: present, gray: absent). The side dendrogram indicates the hierarchical clustering by plasmid replicons. The color of the annotation bar represents the country where the isolate was collected.

Overall, a total of 26 different plasmid replicons have been identified in isolates belonging to PG5, with 99.2% (248/252 strains) carrying at least one plasmid replicon (Fig. 5; Table S7). Among them, IncFIB(K) was the most common (*n* = 233) detected plasmid replicon, followed by IncN2 (*n* = 40) and IncFIB (pQil) (*n* = 37). The greatest number of plasmid replicons identified in one isolate was 5 (*n* = 1), followed by 4 (*n* = 35), 3 (*n* = 39), 2 (*n* = 84), 1 (*n* = 89), and none (*n* = 4). On average, each isolate in PG5 possessed two plasmid replicons. Only one plasmid replicon (IncFIB(K)) was detected in ARM04 and ARM05 (Table S7). In addition to IncFIB(K), ARM03 and ARM06 also harbored IncFIB(Mar) (found in 10.31% of all PG5 strains), IncFII(K) (found in 3.17% of all PG5 isolates), and IncHI1B (found in 8.33% of all PG5 isolates), further indicating that ARM03 and ARM06 may have acquired additional plasmids.

## DISCUSSION

Genomic surveillance studies of *K. pneumoniae* are scarce in Armenia. Recently, we reported the whole-genome sequencing and analysis of a newly emerged *K. pneumoniae* ST967 recovered from a patient in Armenia (31). Such limited data available for *K. pneumoniae* surveillance, including for ST307, prevent a complete mapping of the global population structure and origins of *K. pneumoniae* emerging in different regions of the world, thereby hindering the design of appropriate interventions.

In this study, we report for the first time the whole-genome sequencing and comparative genomic analysis of MDR *K. pneumoniae* ST307 isolates collected from patients in two different hospitals in Armenia. The phylogenetic comparison showed that the Armenian isolates were closely related to each other, with the maximum SNP distance being 39, indicating clonal relatedness. Time-calibrated phylogenetic analysis estimated that the date of the most recent common ancestor of the Armenian isolates was 2005 (95% CI: 1999–2011). Moreover, a phylogenetic clade consisting of four isolates (SRR9854284, SRR10615702, SRR11460696, and SRR11460688) was found to have a close evolutionary relatedness with Armenian isolates and all carried the integrative and conjugative element ICE*Kp*4. The inferred most recent divergence date between these isolates and the Armenian isolates was 2005 (95% CI: 1999–2011).

Although ARM04 and ARM05 were collected from two different hospitals in 2019, they were closely related, exhibiting identical AMR and virulence genes, as well as plasmid replicon profiles (Table 1). There were only three SNP differences in the core genomes between ARM04 and ARM05, with an accessory genome similarity of 98.85%, which together indicate a high genetic similarity. Our findings suggested that there may have been a cross hospital or community transmission of these *K. pneumoniae* ST307 isolates in Armenia. ARM03, ARM06, and ARM05 were collected from different patients in the same hospital in 2019. Among them, ARM03 and ARM06 showed closer phylogenetic relatedness and had similar AMR and virulence genes, as well as plasmid replicon profiles (Table 1). There was no SNP difference in the core genomes between ARM03 and ARM06, with the accessory genome similarity of 98.93%, indicating clonal and direct patient-to-patient transmission. Moreover, we found that ARM03 and ARM06 acquired additional AMR genes (*dfrA5*, *sul1*, *sul3*, *cmlA1*, *mphA*, *mcr-8.1*, and *aph3-Ia*), virulence factors (*iucABCD-iutA* and *rmpADC*), and plasmid replicons (IncFIB(Mar), IncFII(K), and IncHI1B) based on the genome of ARM05. Through BLAST analysis of these gene regions, we found that some additional acquired AMR genes (e.g., *mcr-8.1*) and virulence factors (e.g., *rmp* and *iuc*) in ARM03 and ARM06 may be located on plasmids. Therefore, we hypothesize that the ST307 isolates circulating in different hospitals in Armenia may have acquired plasmids carrying AMR and virulence genes during their transmission and formed a stable transmission network.

As an emerging clone, *bla*_CTX-M-15_-associated MDR *K. pneumoniae* ST307 has spread widely worldwide (1). Consistent with previously published studies (1), we also detected the ESBL gene *bla*_CTX-M-15_ in all four Armenian strains. Antibiotic susceptibility testing revealed that the Armenian isolates were resistant to 8 (*n* = 1) and 9 (*n* = 3) of the antibiotics tested. Our findings indicate that the ampicillin resistance phenotype in the Armenian isolates can be attributed to *bla*_SHV-28_, the cephalosporin antibiotics (ceftazidime, cefepime) resistance phenotypes can be attributed to the ESBL gene *bla*_CTX-M-15_, the fluoroquinolone antibiotics (norfloxacin and levofloxacin) resistance phenotypes can be attributed to *qnrB1*, the chloramphenicol resistance phenotype can be attributed to *cmlA1*, and the β-lactam antibiotics (piperacillin-tazobactam and amoxicillin-clavulanic acid) resistance phenotypes can be attributed to *bl_a_*_OXA-1_ and *bla*_TEM-1D_. However, we were unable to determine the antibiotic resistance genotype that confer phenotypic resistance to chloramphenicol in ARM04 and ARM05, or intermediate resistance to the aminoglycoside antibiotic amikacin in ARM03, as well as intermediate resistance to imipenem in ARM04 and ARM05. Overall, our findings revealed that there was a certain correlation between the presence of a specific antibiotic resistance gene and its resistance phenotype.

Through Scoary analysis, we identified 13 genes unique to the ARM03 and ARM06 isolates. Among them, eight genes (*YmoA*, *inhA*, *mcr-8.1*, *CopR*, *SasA*, *dgkA*, *sbmC*, and *dap*) were located within the same gene region (16,409 bp in length), which we hypothesize is part of the pKP57-mcr8 plasmid (NCBI accession number: CP088130.1).The gene *mcr-8.1* is associated with colistin resistance, a last-resort drug for combat infections caused by MDR Gram-negative bacteria (38). The first IncFII-type plasmid-mediated colistin resistance gene *mcr-8* in *K. pneumoniae* was identified in isolates recovered from chickens and pigs in China (39). Since then, *mcr-8.1* has been widely reported among *K. pneumoniae* isolates from animals and humans around the world, including China (40, 41), Laos (42), and Bangladesh (43), but none from Armenia. Plasmids are thought to play important roles in the dissemination of *mcr*-type gene. *mcr-8.1* has been found on various incompatibility (Inc) plasmid replicon types, such as IncFII, IncFIA, IncFIB, IncQ, IncR, and IncA/C replicons (40, 41). To our knowledge, this is the first detection of the colistin-resistant gene *mcr-8.1* in *K. pneumoniae* ST307 isolates, and it is also the first report of *K. pneumoniae* isolates carrying *mcr-8.1* in Armenia.

The integrative and conjugative element ICE*Kp* is the most common virulence-associated mobile genetic element in *K. pneumoniae* (14). ICE*Kp* has at least 14 structural variants, all of which carry the *ybt* locus (20). *ybt* is one of the siderophores that mediate iron acquisition, an essential element for bacterial metabolic processes (44). Some studies have reported that *ybt* is associated with invasive infections and ICE*Kp* can strongly influence the pathogenicity of *K. pneumoniae* strains (20). Deletion of ICE*Kp* increased the susceptibility of *K. pneumoniae* to antimicrobials, while the introduction of ICE*Kp* or a plasmid-encoding *ybtPQ* into *Escherichia coli* decreased the susceptibility to a broad range of antimicrobials (18). In this study, we found that all four Armenian isolates carried ICE*Kp*4. BLAST analysis revealed that the ICE*Kp*4 sequence was also present in several *E. coli* strains (e.g., *E. coli* LR890603.1 with similarity: 99%, coverage: 99%). Previous reports have also detected ICE*Kp*4 in *K. pneumoniae* of various sequence subtypes, such as ST15 (45), ST147, and ST11 (46), as well as *Klebsiella aerogenes* (47). Our findings, along with existing literature, suggest that ICE*Kp*4 may be a result of cross-species transmission and that those isolates carrying ICE*Kp*4 may well have increased pathogenicity.

In addition to *ybt*, we detected aerobactin siderophore-encoding gene clusters (*iucABCD-iutA*) and the hypermucoidy locus *rmpADC* in ARM03 and ARM06, with ARM06 showing an incomplete *rmpA*, possibly related to the *de novo* assembly of short reads. The *iucABCD* operon and its cognate ferrisiderophore receptor gene *iutA* encode proteins necessary for aerobactin siderophore biosynthesis and transport (48, 49). Previous studies showed that mutant *iutA* (aerobactin deficient) significantly reduced the survival and growth of *K. pneumoniae* (50). The *rmpA* is associated with the hypermucoidy phenotype, a critical virulence factor in hypervirulent *K. pneumoniae* strains. In addition, *rmpA* also serves as a transcriptional regulator for *rmpD* and *rmpC*, and together, these genes form a single operon (51). The *rmpC* is involved in the upregulation of capsule expression (52), whereas *rmpD* drives hypermucoviscosity independently of capsule biosynthesis (53). Ye et al. found that isolates recovered from 40 patients with liver abscesses carried *iuc*, *iro*, *rmpA*, and *rmpA2*, with 35 isolates harboring at least one virulence plasmid (54). We then conducted a BLAST analysis of the regions containing *iuc* and *rmpA* in ARM03 and ARM06. The results showed that these regions belonged to plasmid pK2044 (NCBI accession number: AP006726.1). Classical hv*KP* isolates are usually susceptible to antibiotics (55). Previous comparative studies suggested that MDR *K. pneumoniae* clones might bring a greater threat to the convergence of hypervirulence and multidrug resistance, since they are more likely to acquire virulence genes than hypervirulent clones are to acquire AMR genes (21, 56, 57). Therefore, we hypothesize that ARM03 and ARM06 may have obtained plasmids carrying *iuc* and *rmp*. The acquisition of virulence plasmids could escalate the burden of emerging pathogens for public health services in Armenia and globally due to potential transmission risk.

The main limitation of this study is that the sample size was too small; however, this is the first whole-genome sequencing analysis of *K. pneumoniae* ST307 recovered from patients in Armenia, and most importantly, it is also the first report of colistin resistance gene *mcr-8.1* identified in *K. pneumoniae* ST307 isolates. Our analysis showed that they belonged to the same genetic lineage and had a common ancestor despite being recovered from patients in two different hospitals. All four isolates were resistant to a wide range of antibiotics and carried the integrative and conjugative element ICE*Kp*4 which possessed *ybt* locus. In addition, ARM03 and ARM06 acquired aerobactin siderophore-encoding gene clusters (*iucABCD-iutA*) and the hypermucoidy locus *rmpADC* (ARM06 had incomplete *rmpA*) which all may be located on a mobilizable plasmid. It was evident that there had been a transmission event, which occurred between the two hospitals either through patients or community members. In addition, during such transmission events, the Armenian *K. pneumoniae* ST307 isolates obtained plasmids carrying virulence and AMR genes and formed a stable transmission within the hospital. Our study highlights the importance of genomic surveillance that would contribute to the global efforts to design interventions for limiting the spread of MDR-hypervirulent *K. pneumoniae*.

## MATERIALS AND METHODS

### Identification, antibiotic susceptibility testing, and whole-genome sequencing

Isolate identification, antibiotic susceptibility testing, and whole-genome sequencing of four *K. pneumoniae* isolates included in this study were performed as described in our previous study (31).

### Phylogenetic relationship construction and accessory genome analysis

Previously reported *K. pneumoniae* ST307 genomes were downloaded from the Pathogenwatch database (58) (https://pathogen.watch). All isolates were aligned to the reference genome (GenBank accession number: CP025143.1) and SNPs were identified using Snippy v.4.6.0 (https://github.com/tseemann/snippy). Gubbins v.2.4.1 (59) was performed to remove the recombination fragments. A maximum likelihood phylogenetic tree was reconstructed using FastTree v.2.1.11 (60), under the GTR + GAMMA model with 100 bootstraps replications and visualized using Evolview (61, 62). The phylogeny was partitioned into robust PGs defined through hierarchical Bayesian clustering using FastBaps v.1.0.6 (63). SNP distance between isolates was calculated by snp-dists (https://github.com/tseemann/snp-dists). Roary v.3.13.0 (64) was used to identify core and accessory genes. According to the presence/absence of all accessory genes, Pearson correlation heatmap was constructed in R v.3.6.2 (R Core Team, https://www.R-project.org). Z-scored normalization was used to determine the significance between accessory gene correlation data.

### Time-calibrated phylogenetics

BEAST v.2.6.3 was used to estimate the divergence time (65). CD-HIT was used to cluster and remove repetitive sequences (>99% identity) with the same host species, country, and collection date for the BEAST analysis (66, 67). The optimal nucleotide substitution model was determined using jModelTest v.2.1.10 (68). The GTR + Gamma substitution model, the relaxed clock log normal model, and the Bayesian skyline coalescent model were used. The Markov Chain Monte Carlo chain was run for 10 million generations, each of which was sampled every 1,000 iterations. Convergence and burn-in values were assessed using the Tracer v.1.7.2. The MCC tree was generated using TreeAnnotator and visualized by FigTree v.1.4.4 (https://github.com/rambaut/figtree).

### Virulence factors, antimicrobial resistance genes, and unique genes

Kleborate v.0.3.0 (24) was used to identify K locus, O locus, AMR, virulence genes, and the integrative and conjugative element ICE*Kp* for each isolate. Plasmid replicon types were assessed by searching the PlasmidFinder database with Staramr v.0.7.2 (https://github.com/phac-nml/staramr). Scoary was utilized to identify unique genes in the Armenian isolates (32). Similarity searches of target sequences was performed using the BLAST service at NCBI (https://blast.ncbi.nlm.nih.gov/Blast.cgi). The genomic comparison was visualized using Easyfig (69).

## Data Availability

The short-read sequencing data were deposited in the ENA database under the run accession numbers ERR9882337 (ARM03), ERR9882340 (ARM04), ERR9882341 (ARM05) and ERR9890754 (ARM06). Individual accession numbers for each *K. pneumoniae* ST307 genome sequencing data used in this study are included in Table S2.

## SUPPLEMENTAL MATERIAL

Figure S1 spectrum.01910-24-s0001.tif Count of AMR genes associated with each antibiotic.

Figure S2 spectrum.01910-24-s0002.tif Comparison of *K. pneumoniae* ST307 phylogenetic group (PG) 5 isolates on the accessory genes profile.

Figure S3 spectrum.01910-24-s0003.tif The ICEKp profiles of *K. pneumoniae* ST307 phylogenetic group (PG) 5 isolates (blue: present, gray: absent).

Legends spectrum.01910-24-s0004.docx Legends for Fig. S1 to S3.

Supplemental tables spectrum.01910-24-s0005.xlsx Tables S1 to S7.

ASM does not own the copyrights to Supplemental Material that may be linked to, or accessed through, an article. The authors have granted ASM a non-exclusive, world-wide license to publish the Supplemental Material files. Please contact the corresponding author directly for reuse.

## References

[1] Wyres KL, Hawkey J, Hetland MAK, Fostervold A, Wick RR, Judd LM, Hamidian M, Howden BP, Löhr IH, Holt KE. 2019. Emergence and rapid global dissemination of CTX-M-15-associated Klebsiella pneumoniae strain ST307 . J Antimicrob Chemother 74:577–581. doi:10.1093/jac/dky492 30517666 PMC6376852

[2] Wang S, Zhao J, Liu N, Yang F, Zhong Y, Gu X, Jian Z, Yan Q, Liu Q, Li H, Li Y, Liu J, Li H, Chen L, Liu W. 2020. IMP-38-producing high-risk sequence type 307 Klebsiella pneumoniae strains from a neonatal unit in China. mSphere 5. doi:10.1128/msphere.00407-20 PMC7333572 32611699

[3] Yang Y, Yang Y, Chen G, Lin M, Chen Y, He R, Galvão KN, El-Gawad El-Sayed Ahmed MA, Roberts AP, Wu Y, Zhong LL, Liang X, Qin M, Ding X, Deng W, Huang S, Li HY, Dai M, Chen DQ, Zhang L, Liao K, Xia Y, Tian GB. 2021. Molecular characterization of carbapenem-resistant and virulent plasmids in Klebsiella pneumoniae from patients with bloodstream infections in China. Emerg Microbes Infect 10:700–709. doi:10.1080/22221751.2021.1906163 33739229 PMC8023600

[4] Sartori L, Sellera FP, Moura Q, Cardoso B, Cerdeira L, Lincopan N. 2019. Multidrug-resistant CTX-M-15-positive Klebsiella pneumoniae ST307 causing urinary tract infection in a dog in Brazil. J Glob Antimicrob Resist 19:96–97. doi:10.1016/j.jgar.2019.09.003 31520809

[5] Davies YM, Cunha MPV, Dropa M, Lincopan N, Gomes VTM, Moreno LZ, Sato MIZ, Moreno AM, Knöbl T. 2022. Pandemic clones of CTX-M-15 producing Klebsiella pneumoniae ST15, ST147, and ST307 in companion parrots. Microorganisms 10:1412. doi:10.3390/microorganisms10071412 35889131 PMC9320316

[6] Baron SA, Mediannikov O, Abdallah R, Kuete Yimagou E, Medkour H, Dubourg G, Elamire Y, Afouda P, Ngom II, Angelakis E, Davoust B, Diatta G, Barciela A, Hernandez-Aguilar RA, Sokhna C, Caputo A, Levasseur A, Rolain J-M, Raoult D. 2021. Multidrug-resistant Klebsiella pneumoniae clones from wild chimpanzees and termites in Senegal. Antimicrob Agents Chemother 65:e0255720. doi:10.1128/AAC.02557-20 34152818 PMC8370229

[7] Dropa M, Lincopan N, Balsalobre LC, Oliveira DE, Moura RA, Fernandes MR, da Silva QM, Matté GR, Sato MIZ, Matté MH. 2016. Genetic background of novel sequence types of CTX-M-8- and CTX-M-15-producing Escherichia coli and Klebsiella pneumoniae from public wastewater treatment plants in São Paulo, Brazil. Environ Sci Pollut Res Int 23:4953–4958. doi:10.1007/s11356-016-6079-5 26782324

[8] Peirano G, Chen L, Kreiswirth BN, Pitout JDD. 2020. Emerging antimicrobial-resistant high-risk Klebsiella pneumoniae clones ST307 and ST147. Antimicrob Agents Chemother 64:e01148-20. doi:10.1128/AAC.01148-20 32747358 PMC7508593

[9] Budia-Silva M, Kostyanev T, Ayala-Montaño S, Bravo-Ferrer Acosta J, Garcia-Castillo M, Cantón R, Goossens H, Rodriguez-Baño J, Grundmann H, Reuter S. 2024. International and regional spread of carbapenem-resistant Klebsiella pneumoniae in Europe. Nat Commun 15:5092. doi:10.1038/s41467-024-49349-z 38877000 PMC11178878

[10] Yoon E-J, Gwon B, Liu C, Kim D, Won D, Park SG, Choi JR, Jeong SH. 2020. Beneficial chromosomal integration of the genes for CTX-M extended-spectrum β-lactamase in Klebsiella pneumoniae for stable propagation. mSystems 5:e00459-20. doi:10.1128/mSystems.00459-20 32994286 PMC7527135

[11] Magobo RE, Ismail H, Lowe M, Strasheim W, Mogokotleng R, Perovic O, Kwenda S, Ismail A, Makua M, Bore A, Phayane R, Naidoo H, Dennis T, Ngobese M, Wijnant W, Govender NP, for Baby GERMS-SA1. 2023. Outbreak of NDM-1- and OXA-181-producing Klebsiella pneumoniae bloodstream infections in a neonatal unit, South Africa. Emerg Infect Dis 29:1531–1539. doi:10.3201/eid2908.230484 37486166 PMC10370860

[12] Loconsole D, Accogli M, De Robertis AL, Capozzi L, Bianco A, Morea A, Mallamaci R, Quarto M, Parisi A, Chironna M. 2020. Emerging high-risk ST101 and ST307 carbapenem-resistant Klebsiella pneumoniae clones from bloodstream infections in Southern Italy. Ann Clin Microbiol Antimicrob 19:24. doi:10.1186/s12941-020-00366-y 32487201 PMC7266126

[13] Habeeb MA, Haque A, Nematzadeh S, Iversen A, Giske CG. 2013. High prevalence of 16S rRNA methylase RmtB among CTX-M extended-spectrum β-lactamase-producing Klebsiella pneumoniae from Islamabad, Pakistan. Int J Antimicrob Agents 41:524–526. doi:10.1016/j.ijantimicag.2013.02.017 23622882

[14] Khedkar S, Smyshlyaev G, Letunic I, Maistrenko OM, Coelho LP, Orakov A, Forslund SK, Hildebrand F, Luetge M, Schmidt TSB, Barabas O, Bork P. 2022. Landscape of mobile genetic elements and their antibiotic resistance cargo in prokaryotic genomes. Nucleic Acids Res 50:3155–3168. doi:10.1093/nar/gkac163 35323968 PMC8989519

[15] Botelho J, Grosso F, Peixe L. 2019. Antibiotic resistance in Pseudomonas aeruginosa - mechanisms, epidemiology and evolution. Drug Resist Updat 44:100640. doi:10.1016/j.drup.2019.07.002 31492517

[16] Ramirez MS, Traglia GM, Lin DL, Tran T, Tolmasky ME. 2014. Plasmid-mediated antibiotic resistance and virulence in Gram-negatives: the Klebsiella pneumoniae paradigm. Microbiol Spectr 2:1–15. doi:10.1128/microbiolspec.PLAS-0016-2013 PMC4335354 25705573

[17] Partridge SR, Kwong SM, Firth N, Jensen SO. 2018. Mobile genetic elements associated with antimicrobial resistance. Clin Microbiol Rev 31. doi:10.1128/CMR.00088-17 PMC6148190 30068738

[18] Farzand R, Rajakumar K, Barer MR, Freestone PPE, Mukamolova GV, Oggioni MR, O’Hare HM. 2021. A virulence associated siderophore importer reduces antimicrobial susceptibility of Klebsiella pneumoniae. Front Microbiol 12:607512. doi:10.3389/fmicb.2021.607512 33584611 PMC7876324

[19] Jati AP, Sola-Campoy PJ, Bosch T, Schouls LM, Hendrickx APA, Bautista V, Lara N, Raangs E, Aracil B, Rossen JWA, Friedrich AW, Navarro Riaza AM, Cañada-García JE, Ramírez de Arellano E, Oteo-Iglesias J, Pérez-Vázquez M, García-Cobos S, Sánchez AMF, Pulido MA, Armas M, Dutch and Spanish Collaborative Working Groups on Surveillance on Carbapenemase-Producing Enterobacterales. 2023. Widespread detection of yersiniabactin gene cluster and its encoding integrative conjugative elements (ICEKp) among nonoutbreak OXA-48-producing Klebsiella pneumoniae clinical Isolates from Spain and the Netherlands. Microbiol Spectr 11:e0471622. doi:10.1128/spectrum.04716-22 37310221 PMC10434048

[20] Lam MMC, Wick RR, Wyres KL, Gorrie CL, Judd LM, Jenney AWJ, Brisse S, Holt KE. 2018. Genetic diversity, mobilisation and spread of the yersiniabactin-encoding mobile element ICEKp in Klebsiella pneumoniae populations. Microb Genom 4:e000196. doi:10.1099/mgen.0.000196 29985125 PMC6202445

[21] Wyres KL, Wick RR, Judd LM, Froumine R, Tokolyi A, Gorrie CL, Lam MMC, Duchêne S, Jenney A, Holt KE. 2019. Distinct evolutionary dynamics of horizontal gene transfer in drug resistant and virulent clones of Klebsiella pneumoniae. PLoS Genet 15:e1008114. doi:10.1371/journal.pgen.1008114 30986243 PMC6483277

[22] Russo TA, Olson R, Fang C-T, Stoesser N, Miller M, MacDonald U, Hutson A, Barker JH, La Hoz RM, Johnson JR, Backer M, Bajwa R, Catanzaro AT, Crook D, de Almeda K, Fierer J, Greenberg DE, Klevay M, Patel P, Ratner A, Wang J-T, Zola J. 2018. Identification of biomarkers for differentiation of hypervirulent Klebsiella pneumoniae from classical K. pneumoniae. J Clin Microbiol 56:e00776-18. doi:10.1128/JCM.00776-18 29925642 PMC6113484

[23] Russo T.A, Marr CM. 2019. Hypervirulent Klebsiella pneumoniae. Clin Microbiol Rev 32:e00001-19. doi:10.1128/CMR.00001-19 31092506 PMC6589860

[24] Lam MMC, Wick RR, Watts SC, Cerdeira LT, Wyres KL, Holt KE. 2021. A genomic surveillance framework and genotyping tool for Klebsiella pneumoniae and its related species complex. Nat Commun 12:4188. doi:10.1038/s41467-021-24448-3 34234121 PMC8263825

[25] Dong N, Yang X, Chan EW-C, Zhang R, Chen S. 2022. Klebsiella species: taxonomy, hypervirulence and multidrug resistance. EBioMedicine 79:103998. doi:10.1016/j.ebiom.2022.103998 35405387 PMC9010751

[26] Hallal Ferreira Raro O, Nordmann P, Dominguez Pino M, Findlay J, Poirel L. 2023. Emergence of carbapenemase-producing hypervirulent Klebsiella pneumoniae in Switzerland. Antimicrob Agents Chemother 67:e0142422. doi:10.1128/aac.01424-22 36853006 PMC10019205

[27] Zhang R, Lin D, Chan EW-C, Gu D, Chen G-X, Chen S. 2016. Emergence of carbapenem-resistant serotype K1 hypervirulent Klebsiella pneumoniae strains in China. Antimicrob Agents Chemother 60:709–711. doi:10.1128/AAC.02173-15 26574010 PMC4704206

[28] Gu D, Dong N, Zheng Z, Lin D, Huang M, Wang L, Chan EW-C, Shu L, Yu J, Zhang R, Chen S. 2018. A fatal outbreak of ST11 carbapenem-resistant hypervirulent Klebsiella pneumoniae in a Chinese hospital: a molecular epidemiological study. Lancet Infect Dis 18:37–46. doi:10.1016/S1473-3099(17)30489-9 28864030

[29] Liu X, Wu Y, Zhu Y, Jia P, Li X, Jia X, Yu W, Cui Y, Yang R, Xia W, Xu Y, Yang Q. 2022. Emergence of colistin-resistant hypervirulent Klebsiella pneumoniae (CoR-HvKp) in China . Emerg Microb Infect 11:648–661. doi:10.1080/22221751.2022.2036078 PMC8896207 35086435

[30] Marr CM, Russo TA. 2019. Hypervirulent Klebsiella pneumoniae: a new public health threat. Expert Rev Anti Infect Ther 17:71–73. doi:10.1080/14787210.2019.1555470 30501374 PMC6349525

[31] Sheng J, Cave R, Ter-Stepanyan MM, Kotsinyan N, Chen J, Zhang L, Jiang T, Mkrtchyan HV. 2023. Whole-genome sequencing and comparative genomics analysis of a newly emerged multidrug-resistant Klebsiella pneumoniae isolate of ST967. Microbiol Spectr 11:e0401122. doi:10.1128/spectrum.04011-22 37022188 PMC10269624

[32] Brynildsrud O, Bohlin J, Scheffer L, Eldholm V. 2016. Rapid scoring of genes in microbial pan-genome-wide association studies with Scoary. Genome Biol 17:238. doi:10.1186/s13059-016-1108-8 27887642 PMC5124306

[33] Vitt S, Prinz S, Hellwig N, Morgner N, Ermler U, Buckel W. 2020. Molecular and low-resolution structural characterization of the Na^+^-translocating glutaconyl-CoA decarboxylase from Clostridium symbiosum. Front Microbiol 11:480. doi:10.3389/fmicb.2020.00480 32300335 PMC7145394

[34] Di Berardino M, Dimroth P. 1996. Aspartate 203 of the oxaloacetate decarboxylase beta-subunit catalyses both the chemical and vectorial reaction of the Na^+^ pump. EMBO J 15:1842–1849. doi:10.1002/j.1460-2075.1996.tb00534.x 8617230 PMC450101

[35] McAnulty MJ, Wood TK. 2014. YeeO from Escherichia coli exports flavins. Bioengineered 5:386–392. doi:10.4161/21655979.2014.969173 25482085 PMC4601484

[36] Lam MMC, Wyres KL, Judd LM, Wick RR, Jenney A, Brisse S, Holt KE. 2018. Tracking key virulence loci encoding aerobactin and salmochelin siderophore synthesis in Klebsiella pneumoniae. Genome Med 10:77. doi:10.1186/s13073-018-0587-5 30371343 PMC6205773

[37] Yang X, Xie M, Xu Q, Ye L, Yang C, Dong N, Chan EW-C, Zhang R, Chen S. 2022. Transmission of pLVPK-like virulence plasmid in Klebsiella pneumoniae mediated by an Incl1 conjugative helper plasmid. iSci 25:104428. doi:10.1016/j.isci.2022.104428 PMC9160755 35663037

[38] Poirel L, Jayol A, Nordmann P. 2017. Polymyxins: antibacterial activity, susceptibility testing, and resistance mechanisms encoded by plasmids or chromosomes. Clin Microbiol Rev 30:557–596. doi:10.1128/CMR.00064-16 28275006 PMC5355641

[39] Wang X, Wang Y, Zhou Y, Li J, Yin W, Wang S, Zhang S, Shen J, Shen Z, Wang Y. 2018. Emergence of a novel mobile colistin resistance gene, mcr-8, in NDM-producing Klebsiella pneumoniae. Emerg Microbes Infect 7:122. doi:10.1038/s41426-018-0124-z 29970891 PMC6030107

[40] Yang C, Han J, Berglund B, Zou H, Gu C, Zhao L, Meng C, Zhang H, Ma X, Li X. 2022. Dissemination of bla_NDM-5_ and mcr-8.1 in carbapenem-resistant Klebsiella pneumoniae and Klebsiella quasipneumoniae in an animal breeding area in Eastern China. Front Microbiol 13:1030490. doi:10.3389/fmicb.2022.1030490 36338046 PMC9627307

[41] Wu B, Wang Y, Ling Z, Yu Z, Shen Z, Zhang S, Wang X. 2020. Heterogeneity and diversity of mcr-8 genetic context in chicken-associated Klebsiella pneumoniae. Antimicrob Agents Chemother 65:e01872-20. doi:10.1128/AAC.01872-20 33046490 PMC7927853

[42] Hadjadj L, Baron SA, Olaitan AO, Morand S, Rolain JM. 2019. Co-occurrence of variants of mcr-3 and mcr-8 genes in a Klebsiella pneumoniae isolate from Laos. Front Microbiol 10:2720. doi:10.3389/fmicb.2019.02720 31849875 PMC6887894

[43] Farzana R, Jones LS, Barratt A, Rahman MA, Sands K, Portal E, Boostrom I, Espina L, Pervin M, Uddin A, Walsh TR. 2020. Emergence of mobile colistin resistance (mcr-8) in a highly successful Klebsiella pneumoniae sequence type 15 clone from clinical infections in Bangladesh. mSphere 5:e00023-20. doi:10.1128/mSphere.00023-20 32161143 PMC7067589

[44] Perry RD, Fetherston JD. 2011. Yersiniabactin iron uptake: mechanisms and role in Yersinia pestis pathogenesis. Microbes Infect 13:808–817. doi:10.1016/j.micinf.2011.04.008 21609780 PMC3148425

[45] Cardoso B, Esposito F, Fontana H, Fuga B, Moura Q, Sano E, Sato MIZ, Brandão CJ, Oliveira FA, Levy CE, Lincopan N. 2022. Genomic analysis of a Kpi (pilus system)-positive and CTX-M-15-producing Klebsiella pneumoniae belonging to the high-risk clone ST15 isolated from an impacted river in Brazil. Genomics 114:378–383. doi:10.1016/j.ygeno.2021.12.007 34923088

[46] Bolourchi N, Shahcheraghi F, Giske CG, Nematzadeh S, Noori Goodarzi N, Solgi H, Badmasti F. 2021. Comparative genome analysis of colistin-resistant OXA-48-producing Klebsiella pneumoniae clinical strains isolated from two Iranian hospitals. Ann Clin Microbiol Antimicrob 20:74. doi:10.1186/s12941-021-00479-y 34688302 PMC8542297

[47] da Silva KE, de Almeida de Souza GH, Moura Q, Rossato L, Limiere LC, Vasconcelos NG, Simionatto S. 2022. Genetic diversity of virulent polymyxin-resistant Klebsiella aerogenes isolated from intensive care units. Antibiotics (Basel) 11:1127. doi:10.3390/antibiotics11081127 36009996 PMC9405322

[48] Yu WL, Ko WC, Cheng KC, Lee CC, Lai CC, Chuang YC. 2008. Comparison of prevalence of virulence factors for Klebsiella pneumoniae liver abscesses between isolates with capsular K1/K2 and non-K1/K2 serotypes. Diagn Microbiol Infect Dis 62:1–6. doi:10.1016/j.diagmicrobio.2008.04.007 18486404

[49] Lee CR, Lee JH, Park KS, Jeon JH, Kim YB, Cha CJ, Jeong BC, Lee SH. 2017. Antimicrobial resistance of hypervirulent Klebsiella pneumoniae: epidemiology, hypervirulence-associated determinants, and resistance mechanisms. Front Cell Infect Microbiol 7:483. doi:10.3389/fcimb.2017.00483 29209595 PMC5702448

[50] Russo TA, Olson R, MacDonald U, Beanan J, Davidson BA. 2015. Aerobactin, but not yersiniabactin, salmochelin, or enterobactin, enables the growth/survival of hypervirulent (hypermucoviscous) Klebsiella pneumoniae ex vivo and in vivo. Infect Immun 83:3325–3333. doi:10.1128/IAI.00430-15 26056379 PMC4496593

[51] Walker KA, Miner TA, Palacios M, Trzilova D, Frederick DR, Broberg CA, Sepúlveda VE, Quinn JD, Miller VL. 2019. A Klebsiella pneumoniae regulatory mutant has reduced capsule expression but retains hypermucoviscosity. MBio 10:e00089-19. doi:10.1128/mBio.00089-19 30914502 PMC6437046

[52] Mike LA, Stark AJ, Forsyth VS, Vornhagen J, Smith SN, Bachman MA, Mobley HLT. 2021. A systematic analysis of hypermucoviscosity and capsule reveals distinct and overlapping genes that impact Klebsiella pneumoniae fitness. PLoS Pathog 17:e1009376. doi:10.1371/journal.ppat.1009376 33720976 PMC7993769

[53] Walker KA, Treat LP, Sepúlveda VE, Miller VL. 2020. The small protein RmpD drives hypermucoviscosity in Klebsiella pneumoniae. MBio 11:mBio doi:10.1128/mBio.01750-20 PMC7512549 32963003

[54] Ye M, Tu J, Jiang J, Bi Y, You W, Zhang Y, Ren J, Zhu T, Cao Z, Yu Z, Shao C, Shen Z, Ding B, Yuan J, Zhao X, Guo Q, Xu X, Huang J, Wang M. 2016. Clinical and genomic analysis of liver abscess-causing Klebsiella pneumoniae identifies new liver abscess-associated virulence genes. Front Cell Infect Microbiol 6:165. doi:10.3389/fcimb.2016.00165 27965935 PMC5126061

[55] Surgers L, Boyd A, Girard PM, Arlet G, Decré D. 2016. ESBL-producing strain of hypervirulent Klebsiella pneumoniae K2, France. Emerg Infect Dis 22:1687–1688. doi:10.3201/eid2209.160681 27532217 PMC4994372

[56] Zhang Y, Chen C, Wu J, Jin J, Xu T, Zhou Y, Cui P, Chen J, Chen S, Jiang N, Zhang W. 2022. Sequence-based genomic analysis reveals transmission of antibiotic resistance and virulence among carbapenemase-producing Klebsiella pneumoniae strains. mSphere 7:e0014322. doi:10.1128/msphere.00143-22 35546482 PMC9241541

[57] Shankar C, Jacob JJ, Vasudevan K, Biswas R, Manesh A, Sethuvel DPM, Varughese S, Biswas I, Veeraraghavan B. 2020. Emergence of multidrug resistant hypervirulent ST23 Klebsiella pneumoniae: multidrug resistant plasmid acquisition drives evolution. Front Cell Infect Microbiol 10:575289. doi:10.3389/fcimb.2020.575289 33330125 PMC7718023

[58] Argimón S, David S, Underwood A, Abrudan M, Wheeler NE, Kekre M, Abudahab K, Yeats CA, Goater R, Taylor B, Harste H, Muddyman D, Feil EJ, Brisse S, Holt K, Donado-Godoy P, Ravikumar KL, Okeke IN, Carlos C, Aanensen DM, NIHR Global Health Research Unit on Genomic Surveillance of Antimicrobial Resistance. 2021. Rapid genomic characterization and global surveillance of Klebsiella using Pathogenwatch. Clin Infect Dis 73:S325–S335. doi:10.1093/cid/ciab784 34850838 PMC8634497

[59] Croucher NJ, Page AJ, Connor TR, Delaney AJ, Keane JA, Bentley SD, Parkhill J, Harris SR. 2015. Rapid phylogenetic analysis of large samples of recombinant bacterial whole genome sequences using Gubbins. Nucleic Acids Res 43:e15. doi:10.1093/nar/gku1196 25414349 PMC4330336

[60] Price MN, Dehal PS, Arkin AP. 2010. FastTree 2--approximately maximum-likelihood trees for large alignments. PLoS One 5:e9490. doi:10.1371/journal.pone.0009490 20224823 PMC2835736

[61] Zhang H, Gao S, Lercher MJ, Hu S, Chen WH. 2012. EvolView, an online tool for visualizing, annotating and managing phylogenetic trees. Nucleic Acids Res 40:W569–W572. doi:10.1093/nar/gks576 22695796 PMC3394307

[62] He Z, Zhang H, Gao S, Lercher MJ, Chen WH, Hu S. 2016. Evolview v2: an online visualization and management tool for customized and annotated phylogenetic trees. Nucleic Acids Res 44:W236–W241. doi:10.1093/nar/gkw370 27131786 PMC4987921

[63] Tonkin-Hill G, Lees JA, Bentley SD, Frost SDW, Corander J. 2019. Fast hierarchical Bayesian analysis of population structure. Nucleic Acids Res 47:5539–5549. doi:10.1093/nar/gkz361 31076776 PMC6582336

[64] Page AJ, Cummins CA, Hunt M, Wong VK, Reuter S, Holden MTG, Fookes M, Falush D, Keane JA, Parkhill J. 2015. Roary: rapid large-scale prokaryote pan genome analysis. Bioinformatics 31:3691–3693. doi:10.1093/bioinformatics/btv421 26198102 PMC4817141

[65] Drummond AJ, Rambaut A. 2007. BEAST: Bayesian evolutionary analysis by sampling trees. BMC Evol Biol 7:214. doi:10.1186/1471-2148-7-214 17996036 PMC2247476

[66] Li W, Godzik A. 2006. Cd-hit: a fast program for clustering and comparing large sets of protein or nucleotide sequences. Bioinformatics 22:1658–1659. doi:10.1093/bioinformatics/btl158 16731699

[67] Fu L, Niu B, Zhu Z, Wu S, Li W. 2012. CD-HIT: accelerated for clustering the next-generation sequencing data. Bioinformatics 28:3150–3152. doi:10.1093/bioinformatics/bts565 23060610 PMC3516142

[68] Darriba D, Taboada GL, Doallo R, Posada D. 2012. jModelTest 2: more models, new heuristics and parallel computing. Nat Methods 9:772. doi:10.1038/nmeth.2109 PMC4594756 22847109

[69] Sullivan MJ, Petty NK, Beatson SA. 2011. Easyfig: a genome comparison visualizer. Bioinformatics 27:1009–1010. doi:10.1093/bioinformatics/btr039 21278367 PMC3065679

