# Supplementary figures and images for: Emergence of mcr-8.1-bearing MDR-hypervirulent Klebsiella pneumoniae ST307
Source: Microbiol Spectr. 2024 Dec 13;13(2):e01910-24. doi: 10.1128/spectrum.01910-24 (PMC11792491; doi:10.1128/spectrum.01910-24)

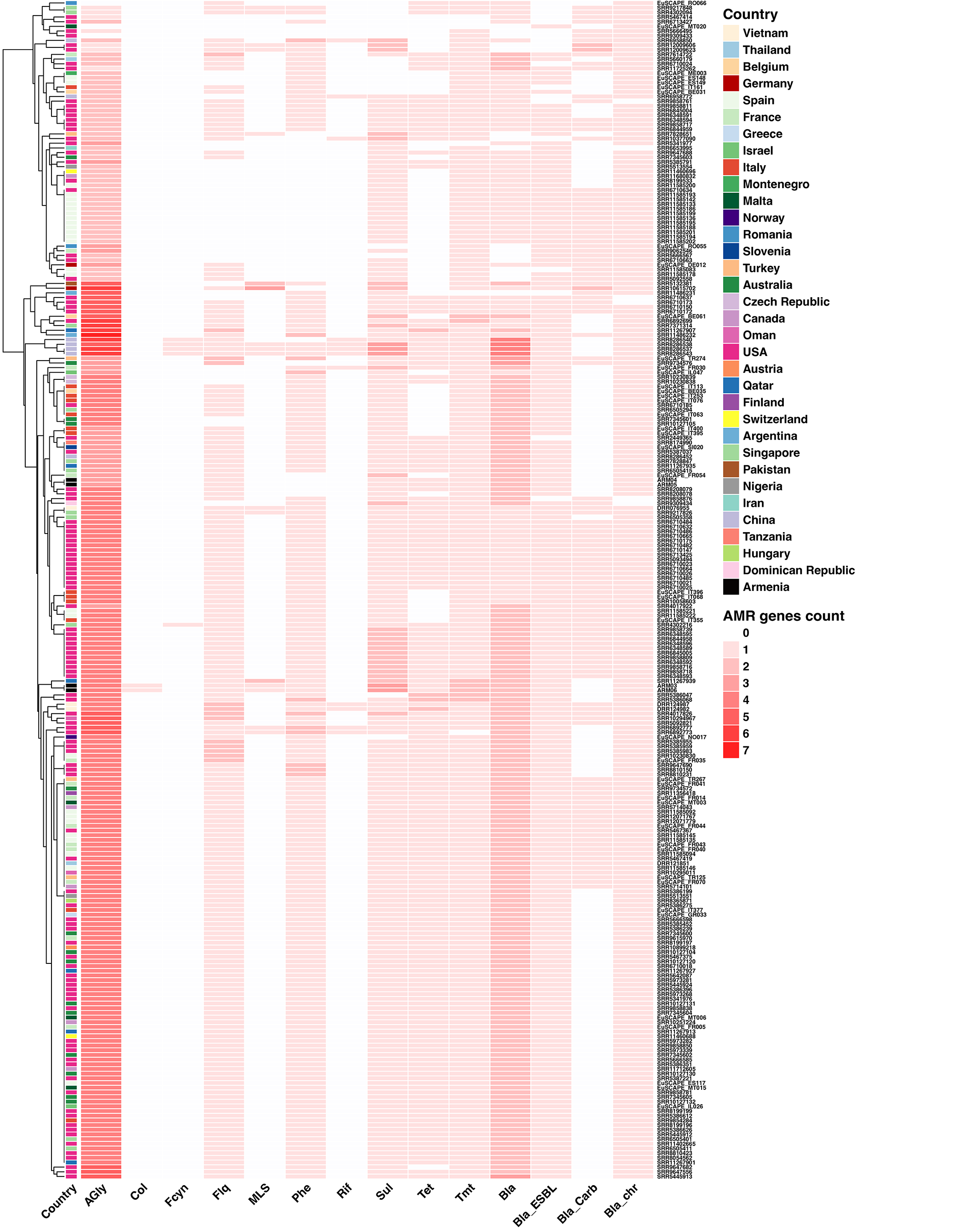

Supplement: Figure S1 — Count of AMR genes associated with each antibiotic. [file spectrum.01910-24-s0001.tif]

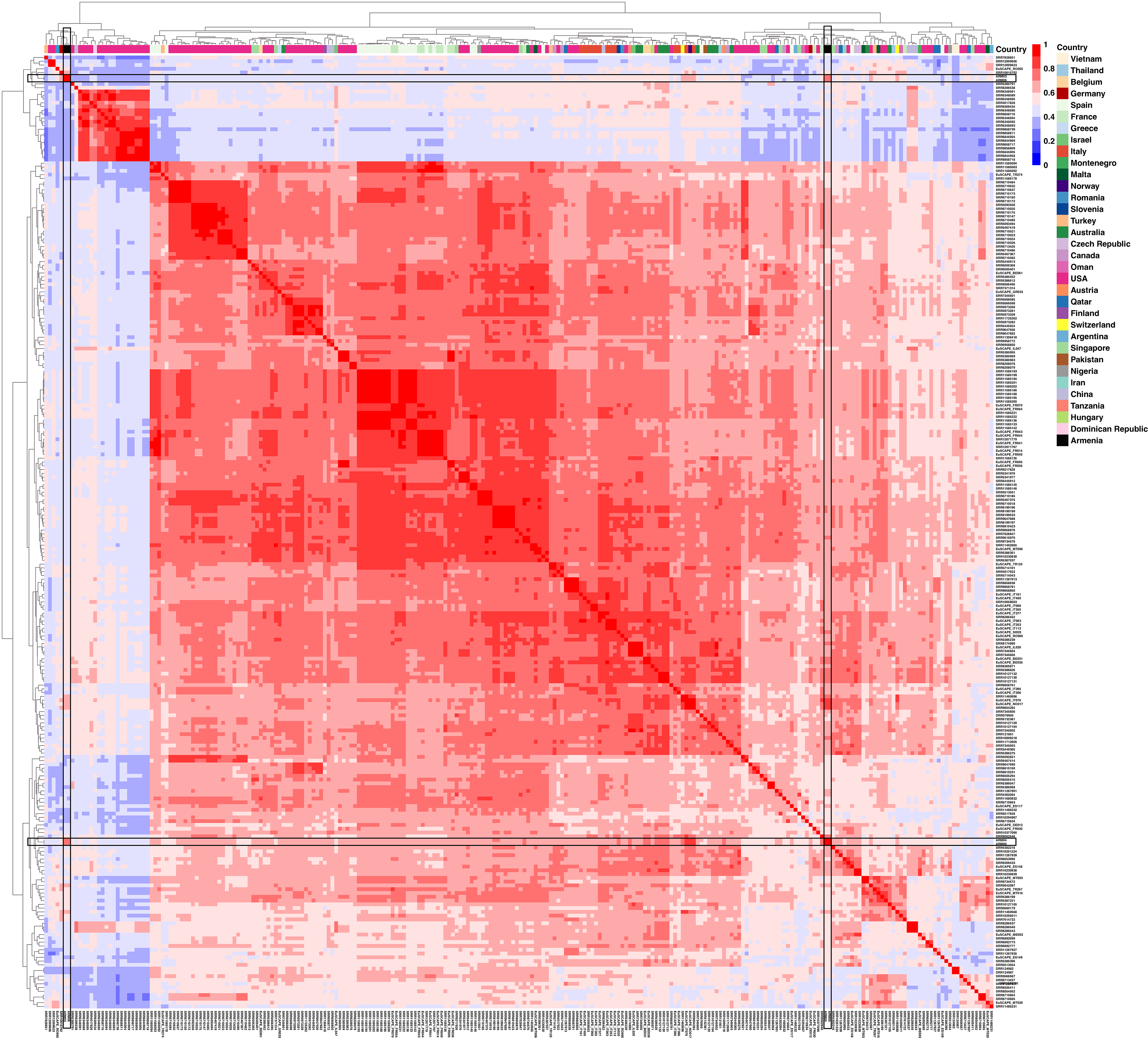

Supplement: Figure S2 — Comparison of K. pneumoniae ST307 phylogenetic group (PG) 5 isolates on the accessory genes profile. [file spectrum.01910-24-s0002.tif]

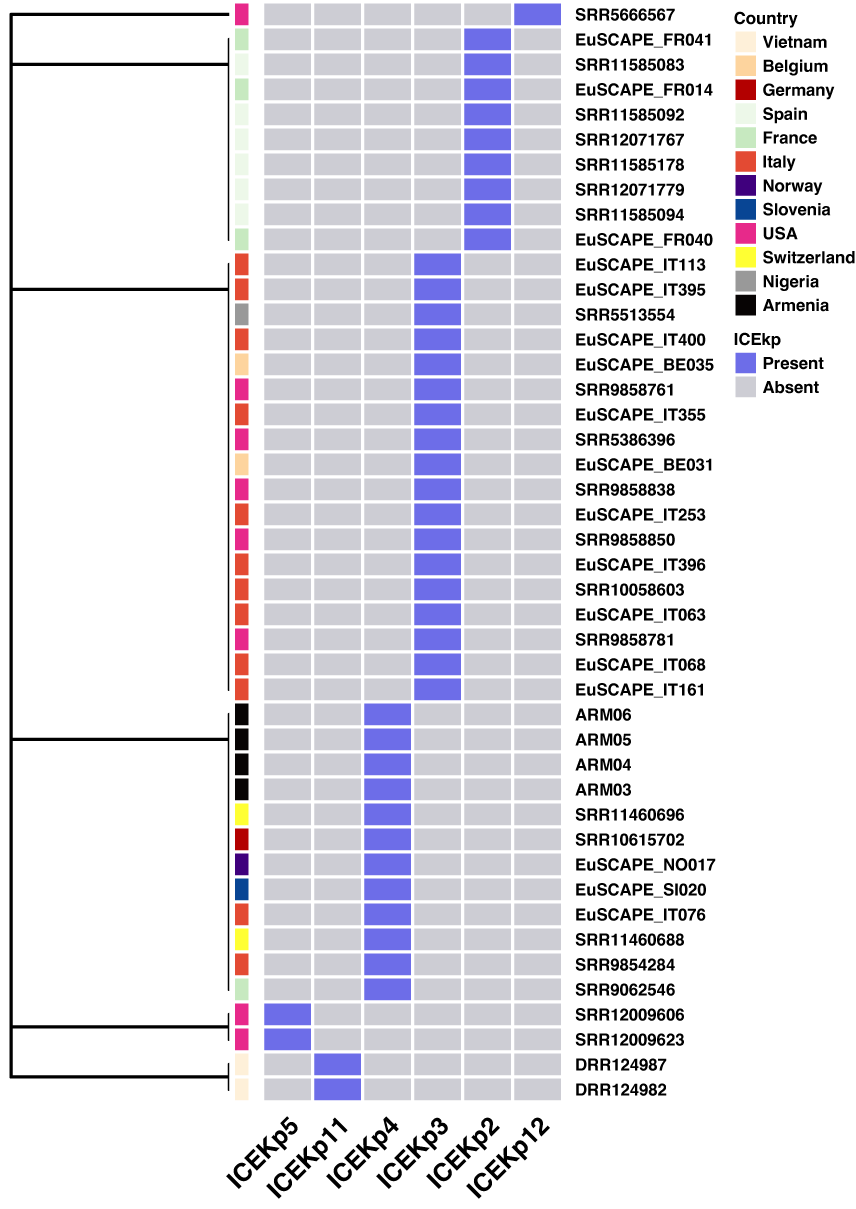

Supplement: Figure S3 — The ICEKp profiles of K. pneumoniae ST307 phylogenetic group (PG) 5 isolates (blue: present, gray: absent). [file spectrum.01910-24-s0003.tif]
