# Supplementary material for: Emergence of mcr-8.1-bearing MDR-hypervirulent Klebsiella pneumoniae ST307
Source: Microbiol Spectr. 2024 Dec 13;13(2):e01910-24. doi: 10.1128/spectrum.01910-24 (PMC11792491; doi:10.1128/spectrum.01910-24)
Supplement: Legends — for Fig. S1 to S3. [file spectrum.01910-24-s0004.docx]

**Figure S1:** Count of AMR genes associated with each antibiotic. The side dendrogram indicates the hierarchical clustering by AMR gene count. The colour of the annotation bar represents the country where the isolate was collected.

**Figure S2:** Comparison of *K. pneumoniae* ST307 phylogenetic group (PG) 5 isolates on the accessory genes profile based on absence (0) or presence (1) matrix. The heatmap shows the accessory genes correlation for all isolates, where red indicates a high correlation and blue indicates a low correlation. The top and side dendrograms indicate the hierarchical clustering by accessory gene content. The colour of the annotation bar represents the country where the isolate was collected. The black box represents the Armenian isolates.

**Figure S3:** The ICE*Kp* profiles of *K. pneumoniae* ST307 phylogenetic group (PG) 5 isolates (blue: present, grey: absent). The side dendrogram indicates the hierarchical clustering by ICE*Kp* structural variants. The colour of the annotation bar represents the country where the isolate was collected.
